# Acknowledgment to Reviewers of *Micromachines* in 2020

**DOI:** 10.3390/mi12020106

**Published:** 2021-01-22

**Authors:** Micromachines Editorial Office

**Affiliations:** MDPI AG, St. Alban-Anlage 66, 4052 Basel, Switzerland

Peer review is the driving force of journal development, and reviewers are gatekeepers who ensure that *Micromachines* maintains its standards for the high quality of its published papers. Thanks to the cooperation of our reviewers, in 2020, the median time to first decision was 13 days and the median time to publication was 32 days. The editors would like to express their sincere gratitude to the following reviewers for their precious time and dedication, regardless of whether the papers were finally published:
Abali, Bilen EmekLiu, Shih-KunAbbasi, Qammer H.Liu, TingyiAbbasi, ZahraLiu, Tzong-shiAbdel Fattah, Abdel RahmanLiu, YingangAbdolrazzaghi, MohammadLiu, ZhixiaAbdolvand, RezaLivreri, PatriziaAbdulkhaleq, AhmedLlobet, JordiAbe, EisukeLo Faro, Maria JosèAbed, Abdel Illah ElLo, Kai-YinAbedini-Nassab, RoozbehLonghi, Patrick EttoreAbidi, KhalidLópez De Lacalle, Luis NorbertoAccardo, AngeloLópez, Ana J.Adrover, AlessandraLópez-Hernández, Luz BereniceAghakhani, AmirrezaLopez-Santos, OswaldoAhadian, SamadLosada, Diego PérezAhmadivand, ArashLova, PaolaAhmmed, K.M. TanvirLovchik, RobertAhnood, ArmanLu, JianAhsan, Dr. Md. ShamimLu, JunpengAkbari, MohsenLu, RuochenAkbaridoust, FarzanLu, YangAkib, ShatirahLuan, Pi-GangAkinsolu, Mobayode O.Luciano, GiorgioAl Sarawi, SaidLucklum, RalfAlabrudziński, SławomirŁukianowicz, CzeslawAl-ajrash, Saja M NabatLumentut, Mikail F.Alam, MehebubLund, AnjaAl-Ameri, TalibLundgren, PerAlapan, YunusLuo, NingqiAlazzam, AnasLuo, QuantianAlbano, GianluigiLuo, TaoAlbarracin, RicardoLuongo, GiuseppeAlden, Dochshanov M.MA, ChaoAleman, BenjaminMa, DaolinAlexandersen, JoeMa, TianjiAlfano, BrigidaMa, YuanAl-Hamidi, YasserMaadi, MohammadAli, HaiderMaamoun, Ahmed H.Alifui-Segbaya, FrankMaasilta, IlariAliyu, AliyuMabbott, SamuelAlkan, BugraMabrook, MohammedAlmeida Júnior, José HumbertoMacaluso, RobertoAlmutairi, ZeyadMacDonald, Niall PatrickAlveringh, DennisMachno, MagdalenaAmarie, DragosMackay, Ruth EAmbrosetti, MatteoMadani, AbbasAmini, HamidMaffettone, Pier LucaAn, Jae-SungMagna, GabrieleAn, Sung JinMahabaduge, HasithaAnand, VijayMahadik, BhushanAndrenko, Andrey S.Maharbiz, Michel M.Anghel, LarisaMaher, HassanAntonopoulos, Christos S.Mahmud, Md ReadulAnufriev, RomanMaikap, SiddheswarApte, NikhilMaire, JérémieAraci, I. EmreMaiuri, PaoloAraujo, DanielMajee, SubimalAraya-Hermosilla, RodrigoMajewski, LeszekArdila, GustavoMajkowska-Marzec, BeataArias-Pardilla, JoaquínMAKINO, TakayukiArigong, BayanerMalakooti, MohammadArima, TakahisaMalecha, KarolArmada, Manuel A.Maletsky, LorinArrabito, Giuseppe DomenicoMaliaris, GeorgiosArscott, SteveMalmstadt, NoahAshraf, NabilMandurrino, MarcoAstakhov, Viktor P.Manoli, KyriakiAsubar, Joel T.Manschwetus, BastianAtaka, ManabuMansouri, AbrahamAtaman, ÇağlarManwar, RayyanAtaollahi, NargesMarcelli, RomoloAusserlechner, UdoMarcos, MarcosAvanaki, Mohammad R. N.Marco-Such, ManuelAversa, AlbertaMariana, IorgulescuAvin, VijayMariani, StefanoAvram, MarioaraMarinesco, StéphaneAxelevitch, AlexanderMarino, MicheleAyuso, José MaríaMarkopoulos, Angelos P.Azadmanjiri, JalalMarkos, ChristosAzizi, EbrahimMarkowski, KonradBabaei, AlirezaMarkowski, Piotr M.Babahosseini, HesamMarpu, Sreekar BabuBaccarani, GiorgioMarques, Manuel Joaquim BastosBadescu, GabrielMarquette, Christophe A.Bae, Hyung D.Marras, AlexanderBae, Sang WonMarrocco, ValeriaBagolini, AlviseMARS, KamelBahcecioglu, GokhanMarszalek, MartaBahi, SlimMartel, SylvainBai, ShiMartel-Foley, JosephBaigmohammadi, MohammadrezaMartin, AndréBailey, NicolaMartin, IrinaBalamurugan, RathinamMartínez López, José IsraelBalasubramanian, PadmanabhanMartínez-Domingo, CarmeBaldas, LucienMartinez-Rivas, AdrianBalme, SébastienMartin-Martinez, JavierBalsamy Kamaraj, AbishekMartino, LucaBamiedakis, NikosMartín-Sánchez, JavierBandara, SumithMarton, MarianBanerjee, AishwaryadevMartucciello, NadiaBanerjee, WritamMaruo, ShojiBañón, FermínMastrangeli, MassimoBao, Jing-fuMateo, DiegoBarbato, MarcoMatetić, MajaBarbosa, JoaquimMathew, BobbyBarfidokht, AbbasMathwig, KlausBarradas, Filipe M.Matos, João NunoBartecki, KrzysztofMatsuda, YuBarth, SvenMatsunaga, DaikiBartolomeo, Antonio DiMauk, Michael G.Bascom, Carlisle S.Maybeck, VanessaBasile, VitoMazzi, AlbertoBastide, StéphaneMcCarron, PaulBatikh, AhmadMcGrath, DanielBattabyal, ManjushaMeacci, ValentinoBecker, AaronMeagher, RobertBehbahani, Amir HosseinMebarek Oudina, FatehBehera, KartikMeda, AliceBelavič, DarkoMedina, LiorBelić, DomagojMeen, Teen-HangBellantone, VincenzoMehrpouya, MehrshadBenedikovic, DanielMei, LanjuBenéitez, Nuria TeigellMelayi, Kiran RajBenhassine, MehdiMelnykowycz, MarkBenneker, AnneMelvin, AdamBeranek, LadislavMendes, Joana CatarinaBerdichevsky, YevgenyMeng, ChaoBeris, AntonyMeng, XinBernacki, KrzysztofMenon, Nishanth VenugopalBernardini, SandrineMenzel, StephanBertana, ValentinaMerenda, AndreaBerto, MarcelloMeroni, SimoneBertsch, ArnaudMerrin, JackBettazzi, FrancescaMia, MozammelBevacqua, Martina TeresaMichel, AndreaBhalla, NikhilMicheli, LauraBhatia, DivijMicheloni, RinoBianco, StefanoMicic, MiodragBieda, MagdalenaMikhaylov, AlexeyBishop, Greg W.Miki, NorihisaBiswas, ChandanMilano, GianlucaBlanche, Pierre-alexandreMillithaler, Jean-FrançoisBober, MariuszMin, Jun-HongBobyr, MaximMin, RuiBoda, Sunil KumarMinerick, AdrienneBodkhe, SampadaMinh, Le VanBognár, GabriellaMirabelli, PeppinoBohinc, KlemenMiranda, AdelaideBöhm, MichałMiranda, Enrique A.Bolivar, JuanMiranda, João MárioBombelli, PaoloMiruszewski, TadeuszBoncel, SlawomirMishra, Anand KumarBonyár, AttilaMishra, AvanishBorboni, AlbertoMishra, Manish KumarBorca, CameliaMishra, NeerajBorghi, FrancescaMistewicz, KrystianBorza, Paul NicolaeMitchell, John S.Botti, SabinaMitrašinović, Aleksandar M.Boujo, EdouardMitsui, ToshiyukiBourouina, TarikMiyata, ShogoBoutayeb, HalimMiyauchi, AkihiroBouwer, James C.Mobbili, GiovannaBožek, PavolMobed-Miremadi, MaryamBragheri, FrancescaMobini, SahbaBrandl, MartinModica, FrancescoBrandus, Catalina-AliceMoghimi, MohammadBravaix, AlainMohammad, MahdaviBrenker, JasonMohd-Yasin, FaisalBriois, PascalMohsen, Marani BarzaniBrogan, JoelMolina-Lopez, FranciscoBrox, MaríaMondal, SudipBrunet, Anton GuimeràMonteleone, SalvatoreBrunet, PhilippeMoon, Byeong-UiBudaszewski, DanielMoosazadeh, MahdiBudzier, HelmutMoradi-Dastjerdi, RasoolBuencuerpo, JerónimoMoramarco, VincenzoBulic, PatricioMoretti, GiacomoBurckhardt, ChristophMori, NobuyaBurugupally, Sindhu PreethamMoshiri, MandanáBuryska, TomasMossi, KarlaButt, M. A.Mouradian, SaraCaballero, DavidMouralova, KaterinaCadore, Alisson RMovafaghi, SanliCahan, RivkaMoyseowicz, AdamCai, ZhenningMu, Luye (Mary)Caiwei, ShenMu, XuanCalabrò, FrancescoMukhin, NikolayCalahorra, YonatanMunteanu, Florentina-DanielaCalaza, CarlosMurari, KrishnaCaligiuri, VincenzoMuravyev, Nikita V.Calin, BogdanMurugan, N. ArulCama, JehangirMuzzio, NicolásCameron, DavidMyasoedova, Tatiana N.Camino, FernandoMyers, OliverCampbell, Joe C.Nabavi, SeyedfakhreddinCampbell, JohnNabki, FredericCampbell, KrisNadolny, KrzysztofCampos, Camila D. MadeiraNagai, MoetoCanfield, BrianNagato, KeisukeCao, JiangNaghdi, SamiraCao, JianyunNagl, StefanCao, LinNagy, GyulaCao, XudongNaito, ToyohiroCao, YunqiNajam, Syed FarazCapaldo, PietroNakagawa, TetsuyaCapochichi-Gnambodoe, MartineNakamoto, HiroyukiCardoso, José RobertoNakano, MichihikoCare, AndrewNakayama, YasuyaCarretero, LuisNan, KewangCarullo, GabrieleNaris, SteryiosCasalino, MaurizioNashimoto, YujiCasalino, MaurizioxNasiri, NoushinCasals Terre, JasminaNasiri, RohollahCasas Bedoya, AlvaroNassiopoulou, AndroulaCatarino, SusanaNassoy, PierreCauwe, MaartenNath, SubhasisaCavina, NicolòNatu, RuchaCea Mingueza, PilarNauli, Andromeda M.Cecconi, CiroNavas-Gonzalez, RafaelCedeño, David L.Navid, KnejadCelano, UmbertoNavlakha, NupurCerbu, CameliaNdukaife, Justus ChukwunonsoCerqueira, LauraNedil, MouradCeyssens, FrederikNeethirajan, SureshChagnon, GregoryNěmec, HynekChakraborty, JoyrajNesterov-Mueller, AlexanderChalyan, TatevikNestler, AndreasChan, Leanne Lai-HangNewland, BenChan, PakNgamson, BongkotChandrahalim, HengkyNgo, Son I.Chandrasekar, HareeshNguyen, TrieuChang, BoNguyen, Tuan-KhoaChang, Liann-BeNiemczewska-Wojcik, MagdalenaChang, Yao-FengNiezabitowski, MichałChang, Yuan-JenNikitenko, AnatoliiChangizian, PooyanNiklaus, FrankChappel, EricNikolaos Pagonis, DimitriosCharkhkar, HamidNikolka, MarkChaubey, SaurabhNilghaz, AzadehChaudhari, AkshayNing, LiqunCheang, U KeiNiu, RanCheepu, Murali MohanNivas, Jijil JJChen, ChaonaNivedita, NiveditaChen, ChengpengNiwano, MichioChen, ChihchenNomura, ShintaroChen, ChuantongNonino, CarloChen, Chun-LiangNosrati, RezaChen, Jung-YaoNunes, LuisChen, Jyh-JianNuñez, NeftaliChen, KaikaiO’Mahony, ConorChen, LibenO’Mullane, AnthonyChen, Lung-ChienObraztsov, Alexander N.Chen, MingzhouObrist, DominikChen, MuyuanOchiai, MasayukiChen, Pang-ShiuOgundile, Olayinka O.Chen, PengyuOgunjimi, Abayomi T.Chen, Pin-ChuanOh, Il-KwonChen, QianOh, JonghyunChen, QiushuOh, TeresaChen, ShutingOhlckers, PerChen, Ting-HsuanOhta, SatoruChen, WeijianOkamoto, SatoruChen, XiangfanOlanrewaju, AyokunleChen, XiangzhongOlariu, Marius-AndreiChen, XinyuanOlga, GuselnikovaChen, YawenOliveira, VítorChen, Yu-ChihOoi, Chin HongChen, Yung-YuOrazbayev, BakhtiyarChen, ZeyuOrazi, LeonardoCheneler, DavidOrbach, RonCheng, ChengOrtega-Casanova, JoaquínCheng, Huanyu (Larry)Oßwald, KaiCheng, Kuang-WeiOstadhassan, MehdiCheng, WeiboOta, NobutoshiCheng, YaOtomański, PrzemysławCheng, Yang-TseOuerghi, AbdelkarimCheng, ZhongYangOwens, Róisín M.Chernyi, SergeiP. Blanchard, JamesChi, Yu-JenPadmanabhan, JagannathChiadò, AlessandroPaek, JungwookChiang, Ya-YuPagáč, MarekChinappi, MauroPagaduan, Jayson V.Ching, Congo Tak ShingPaganin, DavidChing, Tak-ShingPagliara, StefanoChiolerio, AlessandroPagliarini, GiorgioChiriacò, Maria SerenaPaiè, PetraChizhik, AlexanderPajic, BojanCho, Cheong-WeonPal, JitendraCho, Il-HwanPalade, TudorCho, KyungjunePalamà, IlariaCho, Young-hakPalego, CristianoChoi, DukhyunPalevicius, ArvydasChoi, In SungPalm, RainerChoi, Jane RuPan, FeiChoi, JungilPan, Li-ChernChoi, SeongHoPan, YangangChoong, Yu Ying (Clarrisa)Pan, Yong-LeChou, NamsunPan, ZeyuChow, Hwang-CherngPan, Zhuo-HuaChow, Yu-TingPanagiotis, BousoulasChowdhury, FarhanPanda, BiranchiChowdhury, MohamedPanda, Jitendra NarayanChowdhury, Reaz APandey, SantoshChowdhury, SazzadurPandraud, GregoryChristoffersson, JonasPang, FufeiChristol, PhilippePanneerselvam, RajapandiyanChronopoulos, Spyridon K.Pantea, CristianChuang, ChiashainPanuccio, GabriellaChung, Meng TingPaoletti, PaoloChung, MinhwanPapageorgiou, Dimitrios P.Chung, Wan KyunPapalitsas, ChristosChung, YoonyoungParada Medina, RaúlChunxu, MaoPardy, TamasCICOIRA, FabioParia, DebadritaČiplys, DaumantasParida, KaushikCisotto, GiuliaPark, ChoongbaeCivera, MarcoPark, Deok-YongClegg, John R.Park, Hye-JeongClement, MartaPark, Hyeon-CheolCocq, KévinPark, InsuCoello, JuanaPark, JongchanCojoc, DanPark, JunyongCollins, ScottPark, Ki-BumColusso, ElenaPark, Kwan KyuCompany, PedroPark, MinConnolly, James P.Park, MyunghwanConstantin, Gabriel-AlexandruPark, Sang MinConstantinou, IordaniaPark, SunggookConstas, StylianiPark, SungsuCooke, MeganPark, WookCooke, MichaelParra Boronat, LorenaCooksey, Gregory A.Partovi-Azar, PouyaCoppola, SaraParus, ArkadiuszCordill, MeganPatel, SaurinCorrea, DavidPatil, MadhavCorreia, José HiginoPatil, Samadhan BhaulalCorrielli, GiacomoPatra, Saroj KantaCorrigan, NathanielPatrick, ErinCosentino, LuigiPatti, AlessandroCoskun, Ahmet F.Paturzo, MelaniaCosoli, GloriaPaul, SatyamCosta, FrancoPawlowski, AndrzejCosta, PedroPecunia, VincenzoCosta, TiagoPeiner, ErwinCostantini, MarcoPeng, ChangCostanzo, FrancescoPeng, ChuangCotas, CarlaPercoco, GianlucaCottet, JonathanPerec, AndrzejCourtenay, AaronPereira, JoãoCox, TimothyPereira, RubenCrane, NathanPérez, Alberto T.Crescenzi, RoccoPerrier, PierreCritello, Davide C.Persson, Karl-MagnusCronin-Golomb, MarkPervej Jahan, MuhammadCrupi, GiovanniPesch, Georg R.Cuckov, FilipPethig, RonaldCuerno-Rejado, CristinaPetousis, MarkosCui, FeiyunPETRARIU, AdrianCui, WeiPetruska, AndrewCulmer, PetePetryshynets, IvanCummins, GerardPetsagkourakis, Postdoc IoannisCunha, TelmoPezzimenti, FortunatoCzogalla, AleksanderPfattner, RaphaelD’Avino, GaetanoPham, PhuongD’Emilia, GiulioPhan, Hoang-PhuongDai, JingPhan-Quang, Gia ChuongDai, YanweiPhilipp, PatrickDai, YifanPhilipsen, HaroldDalla Betta, Gian-FrancoPhua, Eric J.R.Dalmoro, AnnalisaPiano, MartinaDaltorio, KathrynPiatkevich, Kiryl D.Damanpack, A. R.Picollo, FedericoDandin, Marc P.Piekarz, IlonaDaneshazarian, RezaPierangeli, DavideDang, Phuoc VinhPieri, FrancescoDang, Thai GiangPingarrón, JoséDanielli, AmosPinho, BrunoDanlée, YannPinho, DianaDardano, PrincipiaPires, Ivan Miguel SerranoDaruwalla, AnoshPiskin, SenolDarwish, MohamedPiyasena, MenakeDas, Nirod KPlaczek, MarekDas, PradipPlatz, DanielDas, Proloy TaranPlech, AntonDas, SangitaPlikusiene, IevaDastan, DavoudPlisko, Tatiana V.Dau, Van ThanhPochard, IsabelleDaus, AlwinPodilchak, Symon K.Dave, Utsav D.Poenar, DanielDavid, Gabriela IuliaPogrebnjak, AlexanderDavidsen, Rasmus SchmidtPolese, DavideDavino, DanielePolini, AlessandroDe Dood, Michiel J.Pollard, Shawn DavidDe Lacalle, Luis N. LópezPolley, NabarunDe Oliveira, Helinando PequenoPomastowski, PawełDeja, MariuszPonomarenko, SergeyDel Giudice, FrancescoPons, PatrickDel Hoyo, JesusPorcu, RobertoDelville, Jean-PierrePospíšilová, MarieDemir, MuslumPotkay, JosephDeng, ShuyanPou, JuanDeng, YanxiangPoulikakos, Lisa V.Denkenberger, DavidPouliopoulos, AntonisDeschaume, OlivierPoumellec, B.Desgranges, LionelPrabaswara, AdityaDestgeer, GhulamPrabowo, Briliant AdhiDevendran, CitsabehsanPradhan, ShantanuDey, KamolPrakash, AnandDi Caprio, GiuseppeProietti, EmanuelaDi Flumeri, GianlucaPrzystupa, KrzysztofDi Franco, CinziaPu, YeDi Masi, SabrinaPuchades, IvanDi Noia, Luigi PioPullano, Salvatore A.Di Pietrantonio, FabioPuna, JaimeDíaz, J.Punov, Plamen BorisovDíaz-Otero, Francisco J.Puschita, EmanuelDíez-Jiménez, EfrénPutkonen, Matti IlmariDijksman, J.F.Putrino, GinoDimartino, SimoneQi, DongchenDing, FeiQi, ZenanDing, JunjunQian, ShizhiDing, SonglinQiang, YuhaoDing, XiaoyunQiao, ChunyuDing, XiongQin, ZhenpengDing, ZhaoyangQiu, GangDinh, ToanQiu, TianDixit, Chandra K.Qiu, ZhenDługosz, AdamQu, ChuangDodun, OanaQu, MuchaoDoeven, EganQuadri, MarikaDoi, ToshiyukiQuagliotti, DaniloDoitrand, AurélienQuang Trung, TranDombek, GrzegorzQuarto, MariangelaDominguez, Miguel A.Quesada, Guillermo LópezDong, BoweiRack, Philip D.Dong, GuifangRackus, Darius G.Dong, XiangyangRadwan-Pragłowska, JuliaDong, ZiyeRafhay, QuentinDonnelly, RyanRahimian, ArdavanDorofeev, Sergey G.Rahman, Mohammad H.Dorp, Dennis VanRahman, MuhibUrDoulamis, IliasRahman, Muhommad AzizurDouroumis, DennisRajan, ParthibanDowrick, ThomasRajput, ShailendraDragoman, MirceaRamadan, Qasem M.Drese, Klaus StefanRamakrishnan, SathishDriesche, Sander Van DenRamallo, Manuel V.Drikakis, DimitrisRamamurthy, ChandarasekaranDu, ChaoweiRamanavicius, ArunasDu, HejunRameau, FrançoisDu, SijunRamesan, ShwathyDuan, GuangwuRamezani, ZeinabDuarte Marafona, JoséRamini, AbdallahDubey, NileshkumarRamirez, CristinaDucobu, FrançoisRamón-Azcón, JavierDudek, Krzysztof K.Ramos, DanielDudek, MichalRamos, VictorDueñas Carazo, SalvadorRamser, KerstinDušek, KarelRana, Abu Ul Hassan SarwarDusny, ChristianRangelow, IvoDziurzanski, PiotrRanzato, EliaDzobo, KevinRao, AshutoshEberle, PatricRao, SanjeevEbrahimi Warkiani, MajidRapp, LudovicEcker, MelanieRaptis, IoannisEconomou, DemetreRasouli, ErfanEdelmann, JanRasskazov, IliaEdgar, James H.Rassõlkin, AntonEdwards, Paul R.Ravigururajan, TiruvadiEgan, Paul F.Ravindra, NuggehalliEgashira, KaiRay, VishvaEgatz-Gomez, AnaReboud, JulienEhrmann, AndreaReddy, M. Siva PratapEiber, CalvinReece, Lisa M.Eickenscheidt, MaxReeder, Jonathan T.Elkaseer, AhmedRees, AndrewElmquist, Randolph E.Refatul Islam, MohammadElsayed, MohannadRega, RominaEmadi, ArezooRego, GasparEngelbrecht, RainerReiche, Christopher F.Engevik, Amy C.Reinhardt, AlexandreEom, KyungsikRen, GuanghuiEramo, VincenzoRen, HaoErdem, EmreRen, XiangErfan, MazenRen, XingangEriksen, René LyngeRen, YuanErrico, VitoRen, YukunErtl, PeterResnik, MaticEscapa, AdrianReuben, JohnEscobedo, CarlosRezaei, ShahedEscobedo, PabloRhee, HuinamEsfahani Monfared, YasharRhiger, DavidEsposito, FlavioRho, Hoon SukEuphrasie, SébastienRho, JunsukEysel-Gosepath, KatrinRiahi, RezaEze, VincentRiaud, AntoineFabisiak, KazimierzRichardson, Andrew G.Fakhfouri, ArmaghanRijckaert, HannesFalcón Casas, IgnacioRiordon, JasonFan, Donglei (Emma)Roberge, Jean-PhilippeFan, XugeRoberts, Robert CFan, YuanchengRodrigues, RaquelFan, ZhaoyanRodriguez, Aurelio HierroFan, ZhongmingRodríguez, IsabelFang, HuiRodriguez, Jennifer N.Fang, RunchenRodriguez, NoelFarago, PaulRogdakis, KonstantinosFarah, NairouzRoh, Kyung-HoFarhangdoust, SamanRoh, YongraeFarshbaf, SepidehRoig-Guitart, JaumeFarshchi Yazdi, Seyed Amir FouadRojas-Cárdenas, MarcosFatouros, Dimitrios G.Romani, PatriziaFattahi, AliRomano, LuciaFavalli, MicheleRomero, Francisco J.Fekr, Atena RoshanRomero, Marta R.Feldman, MartinRoncaglia, AlbertoFelgueiras, HelenaRoohnikan, MahdiFeng, HuichengRosenzweig, DerekFeng, QiangRoshanghias, AliFeng, ShilunRosowski, MarkFeng, YinnianRothbauer, MarioFernandes, Mariana Sofia PeixotoRousseau, LionelFernandes, Tiago G.Rowe, BrianFernandez, SusanaRoy, TribeniFernández-Hernández, JesúsRóżańska, SylwiaFerrari, MicheleRuben, PereiraFerraro, AngeloRubiano, AndresFerraro, DavideRubino, GuidoFerraro, PietroRubio, Maria De La Cruz CardenosaFigueiredo, N. M.Rucki, MiroslawFinkler, AmitRudakova, Natalya V.Fiorenza, PatrickRufer, LiborFirebaugh, SamaraRuffin, PaulFišera, MiroslavRusso, SaverioFitzgerald, NeilRuszaj, AdamFlorian Baron, CamiloRyabchikova, Elena I.Fodor, Petru S.Rydosz, ArturFontana, MarcoRyu Cho, Yu KyoungFord, Michael J.Ryu, HanjunForde, Nancy R.Ryu, JehwangFortuna, LuigiRyu, Yu KyoungFoulds, IanRyynänen, TomiFrancais, OlivierSaadatnia, ZiaFrangi, AttilioSabac, AndreiFranssila, SamiSabarou, HoomanFranz, YohannSabín, CarlosFrenea-Robin, MarieSabo, CosminFrense, DieterSachat, Alexandros ElFriedt, Jean-MichelSafa, KasapFroemel, JoergSafonov, IliaFrogley, MarkSaha, BiswajitFu, HailingSai, HiroakiFu, KunSaid, Ibrahim A.Fujino, MasahisaSaijo, YoshifumiFujioka, HidekiSakai, KenjiFukuba, TatsuhiroSakai, ShigekiFukui, TomohiroSakthivel, KogularasuFurlan De Oliveira, RafaelSakthivel, RajalakshmiFutai, NobuyukiSałaciński, TedeuszGadd, MatthewSalaoru, IuliaGadjanski, IvanaŠalić, AnitaGalante, AngeloSalmon, HugoGale, BruceSalvo, PietroGalletti, ChiaraSamiei, EhsanGamella Carballo, MariaSampedro, CarlosGan, Yong X.Sanchez Galan, JavierGangadharan, RajanSandner, ThiloGao, AnmingSanjairaj, VijayavenkataramanGao, HangSanjou, MichioGao, SongSantiago, KevinGao, YongxiangSantos, AbelGarcia-Garcia, Francisco J.Santos, RafaelGarcia-Granada, Andres-AmadorSarcevic, PeterGaroli, DenisSarles, AndyGarrido, Ignacio OchoaSarris, Ioannis E.Gaudiuso, CaterinaSassi, MohamedGeffroy-Rodier, ClaudeSathyanarayanan, GowthamGeints, Yurii ESaumer, MonikaGen, HashiguchiSavoji, HoumanGeorgiev, YordanSaxena, Krishna KumarGerardino, AnnamariaSaxena, PrateekGerber, DoronSaxena, VishalGesuele, FeliceScarisoreanu, NicuGhannad-Rezaie, MostafaSchaap, AllisonGhibaudo, GérardScheler, OttGhila, LuizaSchiavi, Pier GiorgioGhomian, TaherSchiavone, GiuseppeGhosh, AbhishekSchintke, SilviaGhosh, ArijitSchirhagl, RomanaGiacomozzi, FlavioSchlappi, TravisGiannotti, Marina InésSchneckenburger, HerbertGibout, StéphaneSchneider, Ian C.Gieseler, JanSchneider, MichaelGil Santos, EduardoScholten, KeeGiménez-Gómez, PabloSchönemann, LarsGinestra, Paola SerenaSchroedter, RichardGiorcelli, MauroSedao, XxxGiovannini, GiorgiaSegura, JaumeGkoupidenis, PaschalisSeichepine, FlorentGlazar, VladimirSeigneur, NicolasGłuszewski, WojciechSeki, MinoruGnyba, MarcinSelci, StefanoGoehring, TobiasSelvaganapathy, P. RaviGoergen, Craig J.Semaltianos, Nikolaos G.Golgovici, FlorentinaSen, UddalokGómez, VíctorSeo, JihoonGómez-Pastora, JeniferSeo, SoonminGoncalves, Luís M.Seok, SenhoGong, MaxSeredyński, MirosławGong, ZhixiongSeres, JozsefGonzález, BenitoSergeevich, Komlenok MaximGopalakrishnan, KothandamSergi, Pier NicolaGormley, AdamSerhatlioglu, MuratGórski, FilipSerien, DanielaGotow, TakahiroSerjouei, AhmadGottwald, AlexanderSerpooshan, VahidGrace, CarlSerry, MohamedGraf, MarkusSesen, MuhsincanGrattieri, MatteoSethu, PalaniappanGray, Michael D.Setiono, AndiGrech, IvanSetti, DineshGreen, BrendaSeybold, JonathanGreener, JesseShafiee, AbbasGregori, AlbertoShakeel, HamzaGregory, David AlexanderShalaby, MohamedGrenci, GianlucaShameer, KhaderGrieve, James A.Shamshiri, Redmond R.Griffin, Peter B.Shang, LizhiGriggio, FlavioShang, LuoranGrigoriev, FedorShao, LinboGrisé, FabienShao, PengGrosjean, GalienSharafeldin, MohamedGross, Andrew JSharbati, SamanehGrunwald, RuedigerSharker, Shazid Md.Grützmacher, PhilippSharma, AbhishekGu, HengSharma, BharatGu, YuanSharma, PradeepGuan, XiaoweiSharma, Sunil KumarGüemes, AlfredoShateyi, StanfordGuénolé, JulienShavezipur, Mohammad K.Gueorguiev, Gueorgui K.Shaw, KirstyGuijt, RosanneShehata, NaderGulari, ErdoganShekargoftar, MasoudGulati, ShellyShellard, AdamGuldiken, RasimShen, Chih-HsiungGuler, UlkuhanSheng, JunGullo, MaurizioSheng, XingGumuscu, BurcuShi, JunfengGuo, QiuquanShi, QiongfengGuo, YubingShi, QiuweiGupta, ChiragShi, Xiao-LeiGupta, NirzariShieh, Jia-MinGupta, SiddharthShields IV, C. WyattGurban, Ana-MariaShields, WyattGuzman-Sepulveda, Jose R.Shikida, MitsuhiroHa, Dong SamShimizu, JunHaapala, MarkusShimoi, NorihiroHabib, Md AhasanShimokawa, NaofumiHabrat, WitoldSHIN, Dong-MyeongHadas, ZdenekShivakumar, Vibhav BharadwajHaesler, JacquesShivdasani, Mohit NareshHaidegger, TamasShojafar, MohammadHajjaj, Amal Z.Shoji, KanHakem, NadirShokrani, AlborzHall, Derek M.Shore, PaulHamad, EyadShou, DahuaHamann, MartineShpaisman, HagayHamedpour, VahidShrirao, Anil B.Hamelin, BenoitShubhakar, KalyaHamelmann, FrankShyu, Shiuh-HwaHan, Crystal MSiamidi, AngelikiHan, DaehoonSikorski, WojciechHan, GuebumSilva, Rui FHan, Jin-WooSilvera-Batista, CarlosHan, MengdiSimmons, Craig A.Han, WeiSimoni, FrancescoHannu, JariSimonpietro, AgnelloHänsel, AndreasSimorangkir, Roy B. V. B.Hanyková, LenkaSingh, Ajay VikramHao, GuangboSingh, TejinderHao, NanjingSinha, SunilHao, YongSiska, FilipHappy, HenriSkardal, AleksanderHariri-Ardebili, Mohammad AminSkipwith, Christopher G.Harker, AnthonySkoczypiec, SebastianHärmä, HarriSkoulas, EvangelosHarouaka, RamdaneSkrabalak, GrzegorzHasegawa, TomiichiSkripnyak, Vladimir A.Hashimoto, Ken-yaSlate, AnthonyHassan, EmadeldeenSlatineanu, LaurentiuHassan, Sammer-ulSlouka, ChristophHassanin, HanySlouka, ZdenekHassanzadeh-Barforoushi, AminŚlusarczyk, ŁukaszHawkins, Aaron R.Smith, JosephHayakawa, TakeshiSo, HongyunHe, DenghuiSo, Kwok KanHe, QiyuanSoar, JeffreyHe, TianyiyiSobotta, ŁukaszHeinzel, CarstenSobue, ShinichiHejazian, MajidSocol, MarcelaHelerea, ElenaSohn, Dong KeeHellenkamp, Bjoern DavidSong, DongHemsel, TobiasSong, Yong-Ak (Rafael)Hernández Saz, JesúsSong, YuanpingHernando, JordiSorocki, JakubHernando-Garcia, JorgeSoroush, FariborzHerwan, JonnySortino, MarcoHess-Dunning, AllisonSoto, FernandoHeydari Gharahcheshmeh, MeysamSoucy, Jonathan RHeymann, MichaelSpee, BartHill, DanielSpeidel, AlistairHillis, Andrew J.Spiehl, DieterHirai, YoshikazuSpina, RobertoHirtz, MichaelSpolitis, SandisHisey, Colin LeeSreedharan, SreejeshHobbs, RichardSridharan, AratiHoffmann, AndreasSriram, GopuHopper, RichardSriram, RashmiHorváth, JuditSrivastava, SaurabhHossain, GaffarSriwastva, Mukesh KumarHossan, Mohammad RobiulStano, PasqualeHou, XianghuiStarke, EricHsiao, Hsi LienStaszek, KamilHsiao, Hui-HsinStateczny, Andrzej EugeniuszHsiao, VincentStatsyuk, Natalia V.Hssikou, MohamedStavrakis, StavrosHsu, Chai-HsienStefanik, AndrzejHsu, Wei-LunSteinmann, UlrikeHsue, Albert Wen JengStepanova, EkaterinaHu, AnmingStevens, Corey A.Hu, JingtianStewart, RebeccaHu, MiaoSteyer, DanielHu, NingSthal, FabriceHu, XinghaoStiharu, IonHu, YaxiStobiecka, MagdalenaHuang, BoyangStojanovic, GoranHuang, Cheng-ShengStomeo, TizianaHuang, Chia-HuaStornelli, VincenzoHuang, HanSt-Pierre, LucHuang, Hung JiStrauss, VolkerHuang, Kuo-ChengStreckiene, GiedreHuang, Ming-ShyanStrehle, SteffenHuang, Po-HsunStrle, DragoHuang, RuiningStrunin, DmitryHuang, WeichenStuart, Marc C. A.Huang, WeiminStumpe, JoachimHuang, Wen ChangSu, Guo-DungHuang, Yu-HsiSu, HangHuang, Zhaoran (rena)Suddapalli, Chaitanya KumarHuertas, César S.Suea-Ngam, AkkapolHughes, MichaelSukhov, SergeyHughes-Riley, TheodoreŠumak, BoštjanHui, XiaonanSun, ChengHuignard, Jean-PierreSun, GongchenHuling, JenniferSun, KeyeHummon, MatthewSun, LiangHung, Jung-ChouSUN, LinfengHung, Shao-KangSun, MengHur, JaeSun, Yung-ShinHussain, AqeelSun, Yun-LuHussain, GhulamSun, YuxiangHussain, ManwarSung, Gun YongHwang, Gi-ByoungSurace, RossellaHwang, WanSikSurdo, SalvatoreHyun, Kyung-ASuzuki, SeiyaIbarlucea, BergoiSuzuki, TakaakiIbsen, StuartŠvanda, MilanIitani, KentaSwami, NathanIkehashi, TamioSwenson, MatthewIliescu, CiprianŚwiercz, RafałIlinčić, PetarSypniewska-Kaminska, GrażynaIliopoulos, KonstantinosSzukalski, AdamIllarionov, Yury YuryevichSzulczyński, BartoszIlovitsh, TaliSzydsik, CrispinIlyas, AzharTabassum, ShawanaIm, Hyeon-GyunTabish, TanveerInaba, MasafumiTaboryski, Rafael J.Ingebrandt, SvenTadesse, Melkie GetnetIno, KosukeTagit, OyaInoue, ShuheiTahara, HironobuInserra, ClaudeTahara, TatsukiIoakeimidis, ApostolosTaichi Furukawa, TaichiIonescu, Elena RodicaTait, NiallIqbal, FaisalTakahashi, HidetoshiIrvine, Gordon W.Takahata, TomoyukiIrwin, NicolaTakeishi, NaokiIshihara, RyoTalamona, DidierIshii, DaisukeTamarov, KonstantinIslam, MonsurTambuwala, MurtazaIsmaeel, RandTan, CheemengIsoaho, NooraTan, George ZhuoIsozaki, AkihiroTan, Hsih-YinItaka, KenjiTan, HuaIto, YusukeTan, Ming JenIturri, JagobaTanabe, MasayukiIvanescu, MirceaTanaka, NobuyukiIwata, TatsuyaTandecka, KatarzynaIzquierdo-Fuente, AlbertoTang, ShijunIzumi, YudaiTang, ShiyangJ. Black, BryanTang, Shi-YangJ. Jankiewicz, BartłomiejTang, XinJackson, MarkTani, HirofumiJackson, Mark JamesTaniguchi, JunJackson, NathanTanyeri, MelikhanJadidi, MehdiTao, LiJadwiszczak, JakubTapoglou, NikolaosJae Wook, YangTappa, KarthikJahangir, IfatTappan, Bryce C.Jain, GauravTaran, ElenaJakieła, SławomirTarasov, Anton S.Jalili Firoozinezhad, SasanTasca, FedericoJanaideh, Mohammad AlTatara, RobertJanas, DawidTekes, CoskunJanczyk, GrzegorzTelichko, ArseniiJang, HanminTeo, JeremyJang, JinahTeodorczyk, AndrzejJanos, RudolfTeodorescu, FlorinaJanusas, GiedriusTesta, GenniJasielec, Jerzy J.Thacker, Vivek VijayJavorski Eckert, JonyThennadil, SureshJazaeri, FarzanTheodosiou, AntreasJelonnek, JohnThielemann, ChristianeJen, Chun-PingThorneywork, Alice L.Jen, Yi-JunThorpe, StephenJeng, Jeng-YwanThorsen, ToddJeng, Ming-JerThurgood, PeterJeon, SunghoTien, Wei-HsinJeon, TaeyoonTiggelaar, Roald M.Jeong, Chang KyuTimofeev, Andrey V.Jeong, Heon-HoTimpu, FlaviaJeong, JoonsooTittonen, IlkkaJi, XingchenTiwale, NikhilJia, YanweiToda, MasayaJia, YuToledo, JavierJian, JieTomaiuolo, GiovannaJian, Sheng-RuiTomaszewski, DanielJian, WurongTombelli, SaraJiang, BaoyangTomczyk, WojciechJiang, HuaweiTomer, Vijay K.Jiang, JiekeTomita, SatoshiJiang, LaimingTonello, AlessandroJiang, MingzheTong, WeiJiao, YuqingTorisawa, YusukeJin, ChaoTorres-Sospedra, JoaquínJin, HanbitTottori, NaotomoJin, XiaoliangTrache, DjalalJing, WenwenTran, Minh AnhJing, WumingTran, Van-ThaiJing, XiaTrinh, Kieu The LoanJo, Sae ByeokTrinh-Van, SonJohn, Rohit AbrahamTrohman, Richard G.Johnson, StevenTrotta, GianlucaJokinen, VilleTrujillo De Santiago, GrisselJones, PhilipTryznowski, MariuszJönsson-Niedziolka, MartinTsai, Chia-Hung DylanJoshi, NiravTsai, DavidJoshi, SameerTsai, Hsieh-FuJoshi-Imre, AlexandraTsang, Alan Cheng HouJoung, DaehaTsao, Chung-chenJovic, AleksandarTsekenis, GeorgeJuan, Carlos G.Tseng, Kuo-HsiungJuan-Colás, JoséTseng, Sheng-HsiangJukna, VytautasTseng, Shuo-YenJun, Sang BeomTsoukalas, DimitrisJung, Jae HwanTsukahara, TakahiroJung, Seon YeopTu, MinJuodkazis, SauliusTuohiniemi, MikkoJursich, GregoryTuralchuk, PavelJuska, Vuslat B.Ture, ErdinJyoti Bora, PritomTurini, GiuseppeKabiri Ameri, ShidehTyaginov, StanislavKabiri, Ameri ShidehTzounis, LazarosKacem, NajibUluşan, HasanKaczmarek, MichalUm, Doo-SeungKadel, RajanUm, Han-DonKageshima, YosukeUnnithan, Ranjith RajasekharanKageyama, YoshiyukiUnterlass, MiriamKai, HiroyukiUpham, JeremyKaidatzis, AndreasUrban, GeraldKainz, AndreasUrbikain Pelayo, GorkaKakabakos, SotiriosUrsu, IoanKalkman, JeroenUshakov, NikolaiKalkur, T.S.Vafaei, SaeidKamath, RahulVaikuntanathan, VisakhKameoka, JunVala, JiriKaminska-Chuchmala, AnnaValentinčič, JoškoKamperman, TomValiente, DavidKamyshny, AlexValsaraj, AmithrajKandadai, NirmalaVan Dam, R. MichaelKang, ChanKyuVan Der Sluis, OlafKang, DonghoonVan Rooyen, Isabella JKang, HongkiVaradwaj, PradeepKang, Jeon WoongVaradwaj, Pradeep R.Kang, SaewonVardhan, HarshKang, Seok-WonVardiambasis, IoannisKang, Sung-MinVarga, PalKang, Yang JunVarma, Vijaykumar B.Kannappan, RamaswamyVasic, VesnaKantak, ChaitanyaVasile, SimonaKaplan, AndreyVasilescu, Mircea DorinKar, ShantimoyVasjanov, AleksandrKardel, KamranVassant, SimonKarim, LutfulVauche, RemyKaruppan, Mohan Kumar MuthuVázquez De Aldana, Javier RodríguezKaruppuswami, SaranrajVazquez, Rebeca MartinezKarvelas, EvangelosVecchione, Dr RaffaeleKashaninejad, NavidVelásquez-García, Luis FernandoKashir, AlirezaVelha, PhilippeKasper, MikeVeloso, AntonioKathuria, HimanshuVenediktov, VladimirKato, KimihikoVenkatasubramanian, AnandramKato, MasashiVenneri, FrancescaKaushik, AniruddhaVerdurmen, Wouter P.R.Kavanagh, DeanVerna, AlessioKawahara, TomohiroVerotti, MatteoKawai, TakayukiVetter, ChristianKazazis, DimitrisVicarelli, LeonardoKe, Tung-HueiVidal-Álvarez, GabrielKeating, AdrianViefhues, MartinaKee, ScholtenVijayavenkataraman, SanjairajKeskinbora, KahramanVilé, GianvitoKeyvaninia, ShahramViržonis, DariusKhalaj, OmidVizzini, PriyaKhalil, IslamVocale, PamelaKhamis, HebaVogt, JochenKhan, DiganganaVogt, UlrichKhan, IftekharVohnsen, BrianKhan, Mohammad MonirujjamanVoiculescu, IoanaKhan, UsmanVolgin, Vladimir M.Khang, DahlyoungVolkova, Elena G.Khansur, Neamul HayetVollnhals, FlorianKheyraddini Mousavi, BehnamVolosova, Marina A.Khilwani, RakeshVolpe, AnnalisaKhodadadian, AmirrezaVomero, MariaKhodayari Bavil, AliVoronenkov, VladislavKhoo, Bee LuanVoué, MichelKhoshmanesh, KhashayarVoznyak, YuriKhudiyev, TuralVrančić, DamirKhyam, MohammadW.k., FungKiasaleh, KamranWada, Ken-IchiKiazadeh, AsalWägemann, PeterKIEW, Choon MengWagih, MahmoudKilinc, DevrimWalker, Ross M.Kim, AlbertWalker, Travis W.Kim, Chang-HyunWallentin, JesperKim, Chul MinWalther, ThomasKim, DaewonWaltl, MichaelKim, DaeyoungWan, DiKim, Dong WonWang, AnnaKim, Dong-PyoWang, BaomingKim, Eun KyuWang, BinKim, EunhaWang, ChaoKim, GaramWang, Cheng-ChiKim, GunzungWang, ChunjinKim, Hong NamWang, DanqingKim, HyeminWang, DapengKim, HyeokWang, DingkangKim, Hyun SooWang, HaiyangKim, HyungjinWang, HaoKim, Jang AhWang, HaoranKim, Jeong AhWang, HuaKim, Jeong-ahWang, JiaweiKim, Jin SikWang, JingKim, Jin YoungWang, KaiKim, JinsikWang, KaiyingKim, Jong-hoonWang, LingyunKim, JungkyuWang, LiuKim, KeekyoungWang, MinqiangKim, Ki-ChaiWang, Mu-chunKim, KwangsooWang, PengKim, Min-SeokWang, Qiang (Jason)Kim, MunhoWang, QingpuKim, Myung JunWang, QingQingKim, MyunghoiWang, Shau-ChunKim, SejoongWang, TaoKim, Seok-HoWang, WeiKim, SeokminWang, WenboKim, Soo HyeonWang, YanxingKim, Su-HwanWang, YichunKim, Sun KyoungWang, YingKim, Sung EunWang, YulingKim, Sung HyunWang, ZhenfengKim, Sung-HoonWang, ZhiKim, SungjunWang, ZhongruiKim, Tae-HyungWang, ZhuqingKim, Wook-BaeWasisto, Hutomo SuryoKim, YeonginWatanabe, WataruKim, Yong SikWażny, MariuszKim, Yong-GyooWeaver, Wayne W.Kim, Yun HoWebster-Wood, Victoria A.Kimura, MutsumiWegener, JoachimKioseoglou, GeorgeWęglarski, MariuszKirchner, RobertWei, TiweiKireev, DmitryWeihnacht, ManfredKiyohara, KenjiWeinberg, MarcKliros, George SWeisensee, PatriciaKlos, GunnarWells, LauraKlos, Jaroslaw W.Wen, ChunshengKlyui, Nickolai I.Wen, RuitaoKnapkiewicz, PawelWeng, Yung-JinKnospe, CarlWen-Jeng, HoKo, JinaWenya, WUKobayashi, MakikoWhalley, DavidKoblischka, Michael R.Whipple, Chery A.Kocik, MarekWibowo, DavidKoeper, IngoWillenbacher, NorbertKoh, Kah HowWillert, AndreasKojdecki, Marek AndrzejWilliams, John A.Kokkinos, ChristosWilson, Andrew J.Koledova, ZuzanaWinczek, JerzyKong, Byeong YongWiniarski, TomaszKong, XiangruiWinterhalter, MathiasKonovalov, Sergey V.Witczak, PiotrKonstantinidis, GeorgeWittemann, FlorianKontziampasis, DimitriosWłodarczyk, Paweł P.Koo, ChiwanWojciechowski, SzymonKopparthy, Varun LingaiahWojkiewicz, Jean-LucKopra, KariWojnarowicz, JacekKorkmaz, EmrullahWong, HarrisKos, ŽigaWong, Lai MunKosar, AliWong, Man HoiKöstler, StefanWong, PakKothapalli, Sri-Rajasekhar (Raj)Wong, ZijingKotnala, AbhayWoo, Jong-KwanKotz, FrederikWoodfield, PeterKováč, JaroslavWoon, Wei-YenKowiorski, KrystianWrobel, AndrzejKozłowska, JustynaWrobel, PiotrKraft, ThomasWu, Chih YangKraker, ElkeWu, Hung-ChinKrammer, OliverWu, KaiKrasnoslobodtsev, Alexey V.Wu, LixiangKrieg, MichaelWu, MengxiKrishnarjuna, BankalaWu, NanKrólicka, AgnieszkaWu, WeiKüçüköz, BetülWu, XiaofengKuczmaszewski, JozefWu, XinKudryashov, Sergey I.Wu, ZhiyiKuehne, Alexander J. C.Wuethrich, AlainKugimoto, KoXavier, MiguelKukla, ChristianXia, ZhanboKulinich, SergeiXiang, ChaoqunKulothungan, JothiramalingamXiang, JiwenKumar K B, VinayaXiao, XinKumar, AbhishekXiao, YuanKumar, ArkadeepXiao, ZhigangKumar, ArunXie, ShiyuKumar, BijandraXie, YuliangKumar, MohitXie, ZuotiKumar, PawanXing, GuichuanKumar, RajeshXiong, ZeKumar, SanjeevXiros, NikolaosKumar, ShailabhXU, KaichenKumaresan, YogeenthXu, LeiKumavor, Patrick D.Xu, QinKung, Yu-ChunXu, WeiheKunimine, TakahiroXu, YongKuo, Po-LingYadavali, SagarKuo, Wen-chengYadavalli, Vamsi KKurc, BeataYalikun, YaxiaerKurlyandskaya, Galina V.Yamada, AyakoKurniawan, RendiYamamoto, KenKutin, JožeYamamura, ShoheiKuznetsova, Iren E.Yamashiro, SawakoKwak, Tae JoonYamazaki, HirohitoKwapiszewska, KarinaYan, BingxiKwon, HyukjinYan, JinKwon, Hyuk-YoonYan, WeiKwon, Kye-SiYang, BinKwon, Oh SeokYang, C.-F.Kwon, Seok-JoonYang, GengKyratsis, PanagiotisYang, Hung-WeiLa Rocca, MicheleYang, JieunŁabanowski, JerzyYang, LiangLabuta, JanYang, RuiguoLackowski, MarcinYang, XusanLaezza, AntonioYang, YangLafata, PavelYang, YansongLafratta, ChristopherYang, Ya-TangLaghrouche, MouradYang, YingLaity, Peter RYang, YueLaleh, RadYang, YujiaLam, MailieYang, ZhenchuanLam, RaymondYang, ZhihongLamberti, Vincent E.Yang, ZongyinLane, ChristopherYao, Da-JengLang, WalterYap, Yiing ChiingLanzalaco, SoniaYapici, Murat KayaLarsson, KarinYarali, MiladLatorre-Rey, Alvaro D.Yasa, Immihan CerenLaurenti, MarcoYasukawa, TomonoriLauro, Michele DiYe, FanLautner, GergelyYe, JianchaoLaviano, FrancescoYeager, John D.Lázaro, AntonioYen, Yi-KuangLazarus, NathanYeom, JunghoonLe Borgne, BriceYi-chin, TOHLe Gac, SéverineYilmaz, MehmetLe Moal, PatriceYin, JieLeal-Junior, ArnaldoYo, TanakaLeavesley, DavidYokota, KazumichiLechuga, YolandaYong, Kar WeyLecler, SylvainYoon, Hyeun JoongLeclerc, JulienYoon, Jae WoongLecomte, Jean-SebastienYoon, JinhoLedo, AnaYoon, Woon-HaLee, AlbertYossifon, GiladLee, BongmookYou, Yil-HwanLee, Byoung-SunYu, Chin-PingLee, Byung ChulYu, JiangfanLee, DaeseokYu, KaiyanLee, Gil-YongYu, MengjieLee, GyudoYu, WeizeLee, Hee-JoYu, XiaomingLee, HughYu, YangLee, Hyunjoo J.Yu, ZiyiLee, HyunseopYuan, GuanghuiLee, InheeYuan, HaoLee, JaewooYun, JinHyeonLee, JungchulZabek, DanielLee, KeekeunZaitsev, Boris D.Lee, KyutaeZakrzewska, KatarzynaLee, Myung-JaeZalar, PeterLee, SanghoonZandrini, TommasoLee, Seok JaeZavašnik, JanezLee, SeunghyunZayachuk, YevhenLee, SeungyunZebrev, GennadyLee, Sung SikZeck, GüntherLee, Tae-IkZega, ValentinaLee, Wen-JenZelbst, PamelaLee, Won GuZeljkovic, MilanLee, WonheeZemanek, PavelLee, Yung-chunZeng, HaoLei, JinchengZergioti, IonnaLei, ShutingZerulla, DominicLei, UZetterling, Carl-MikaelLeissner, TillZha, GangqiangLemma, EnricoZhai, ShengjieLentini, LuigiZhang, ChengLeoni, AlfieroZhang, DongzhouLesiak, PiotrZhang, EnxiaLettieri, StefanoZhang, Eric J.Levin, AviadZhang, GuangpingLi, DongyingZhang, HuilongLi, FengZhang, JianLi, HaijunZhang, JihuaLi, HongjianZhang, JunLi, Jin-FuZhang, QiLi, JinganZhang, QiangLi, KezhengZhang, ShimingLi, LihuaZhang, ShixiongLi, MingZhang, ShunpingLi, Ming-YuZhang, TianxiaoLi, PengZhang, WeidongLi, PengzhiZhang, WenjunLi, ShuangmingZhang, XianghuiLi, TaoZhang, XiaoshengLi, TianlongZhang, XinjieLi, WenmingZhang, XumingLi, XiangZhang, YaLi, XiaoZHANG, YILi, XinboZhang, YongLi, YangminZhang, Yong-HangLi, YiZhang, YunyanLi, YiyanZhao, ChenglongLi, ZhihongZhao, ChumingLi, ZhikangZhao, HaitaoLi, ZhipengZhao, JielingLi, ZidongZhao, KaiLiang, Yuan-ChangZhao, PingLiao, XinqinZhao, XiaoguangLibra, MassimoZhao, YapuLiedl, GerhardZhao, YuLien, Der-HsienZhao, YulongLietha, DanielZheng, BoLigęza, PawełZheng, JinchuanLim, JiseokZheng, LuLima, IvanZheng, WenshuLima, RuiZheng, YihaoLin, Chih-TingZheng, YuebingLin, Chih-YuanZherdev, Anatoly V.Lin, Chun-HoZhou, BoLin, Der-YuhZhou, JiajingLin, FrancisZhou, JianLin, Jr-LungZhou, KunLin, LinhanZhou, RanLin, T. HZhou, XiangLin, XiaoyouZhou, YuqingLin, YangZhu, BowenLin, YuankunZhu, DazhangLin, ZhiyuanZhu, HongtaoLin, Zuan-TaoZhu, LiangdongLisowski, EdwardZhu, XuanLitvić, MladenZhu, YiboLitwin, DariuszZhu, YingLitzius, KaiZhu, ZijieLiu, BaiyangZhukov, ArcadyLiu, BaojiangZhuo, YueLiu, Cheng-HsienZidek, JanLiu, ChuanZivkovic, VladimirLiu, GuigenZizzari, AlessandraLiu, HeliZolfagharian, AliLiu, KuiZoltán, FeketeLiu, MengmengZou, HaiyangLiu, QiancaoZywica, Grzegorz

